# Legal Evidence–Authority Dissociation in juror judgment: a multistudy reanalysis of memory contamination and AI advice

**DOI:** 10.3389/fpsyg.2026.1956486

**Published:** 2026-10-16

**Authors:** Xiangran Xie

**Affiliations:** School of Law, Tianjin University, Tianjin, China

**Keywords:** artificial intelligence, equivalence testing, evidence evaluation, juror decision-making, legal psychology, misinformation, source monitoring, specification curve

## Abstract

**Introduction:**

Legal decision makers may remember information without using it to update a legal judgment, and may respond differently to the same recommendation depending on its attributed source. LEAD separates these processes into memory-entry, judgment-incorporation, and authority-deference gates.

**Methods:**

We reanalyzed three public, de-identified datasets: one jury-deliberation misinformation study and two preregistered judge-versus-artificial-intelligence sentencing studies. We compared gate-specific estimates while retaining study-specific outcomes and raw units.

**Results:**

In Study 1, pro-prosecution misinformation crossed the memory-entry gate more clearly than the judgment-incorporation gate. In Studies 2 and 3, mitigating case facts produced larger judgment-incorporation effects than authority-source assignment. The authority-source effect was equivalent to zero within a prespecified small-effect bound in Study 2 (source Experiment 1), but remained inconclusive in Study 3 (source Experiment 2).

**Discussion:**

LEAD identifies which process changed and supports cross-gate magnitude comparisons without treating unlike outcomes as identical. This secondary analysis does not estimate behavior in real courts, but it provides a transparent framework for forensic and legal psychology.

## 1 Introduction

Legal fact-finding depends on a psychological chain that is more fragile than the legal record makes it appear. Evidence must be encoded, remembered as coming from the trial rather than from post-event discussion, interpreted in light of a disputed narrative, and translated into a judgment about guilt, credibility, or punishment. A legal proposition therefore has both an epistemic status, because it may or may not support an inference, and a psychological status, because it may or may not be remembered and used by the decision maker. Classic work on the story model of juror reasoning and coherence-based legal judgment shows that fact finders organize evidence into narratives rather than passively adding independent pieces of information

(Pennington and Hastie, 1992; Carlson and Russo, 2001; Simon and Read, 2023). This makes legal decision-making a particularly useful setting for testing whether memory, evidence use, and authority deference move together or dissociate.

Misinformation research provides one route into this problem. Eyewitness and post-event information studies have shown that memory reports can be altered by later information, warnings, and source-monitoring demands (Loftus, 2005; Jacoby, 1999; Blank and Launay, 2014). More recent work emphasizes that misinformation effects are not a single mechanism: belief updating, source reliability, causal explanation, and correction can each shape whether false information persists (Ecker et al., 2011, 2015, 2022). In a jury context, the critical question is not only whether a person later reports a contaminated detail. It is also whether the contaminated detail is treated as legally probative evidence when the person updates a conclusion about a defendant or a complainant. The recent jury-deliberation experiments by Cullen et al. (2024) make this distinction empirically visible: misinformation introduced during deliberation affected source memory, but its effects on legal decision measures were not uniform across studies.

Recent forensic-psychology work makes the same problem more concrete. Eyewitness confidence can be shaped by interview structure, false-memory susceptibility can vary with self-relevance, and legal professionals do not always have accurate beliefs about false-memory risks (Caso et al., 2024; Wang et al., 2024; Houben et al., 2024). Work on false confessions and courtroom directions further shows that legal conclusions depend on how decision makers interpret a memory, statement, or instruction within a procedure (Welner et al., 2024; Wieberneit et al., 2026). A common numerical scale can make effect sizes comparable. The problem arises when one response is treated as evidence for several processes, such as assuming that a remembered detail was also used to change a legal judgment.

The same separation is needed when legal decision makers receive advice from judges or artificial intelligence systems. “AI in courts” covers several distinct uses. Systems may support legal research, document review, case administration, transcription, translation, outcome prediction, or a human decision rather than replace a judge (European Commission for the Efficiency of Justice, 2018). The information supplied to a system depends on its function. A document tool may process judicial decisions or filings, whereas a criminal-risk tool may use structured information such as age and criminal history (Dressel and Farid, 2018). These uses raise questions about transparency, fairness, and meaningful human control. They do not imply that a trial transcript is always supplied to an AI system.

The sentencing studies reanalyzed here tested a narrower situation. Participants were told that an experienced judge and an AI trained on case law received the same defendant background, criminal-record information, and mitigating circumstances (Watamura et al., 2025). No working AI analyzed a trial transcript. The manipulated factor was which authority, judge or AI, recommended prison when their recommendations conflicted. Research on algorithm aversion and appreciation suggests that responses to such a label depend on the task, perceived affective capacity, error visibility, and decision domain (Logg et al., 2019; Castelo et al., 2019; Araujo et al., 2020). A concrete comparison illustrates the distinction: adding domestic-violence mitigation changes the facts of the case, whereas swapping the judge and AI labels changes only who recommends prison. These changes need not affect sentencing to the same degree.

Legal AI benchmarks reinforce the need for precise outcomes. LegalBench, LawBench, and recent evaluations separate doctrinal knowledge, textual analysis, and legal application rather than relying on one accuracy score (Guha et al., 2023; Fei et al., 2024; Mishra et al., 2025). These benchmarks evaluate models, not juror psychology. A juror study instead asks whether factual content changes a legal conclusion and whether the identity attached to a recommendation changes reliance. Large language models can participate in mock legal tasks, but model performance and human authority deference remain different questions (Sun et al., 2025).

The distinction is practically important because legitimacy, expertise, and bias are contested for both human and automated advice. AI-generated media, forensic expert mediation, and AI-supported administration may alter trust before they alter a formal outcome (Iannuzzi et al., 2025; Hefetz, 2025; Krištofík, 2025). Human legal authority is not a bias-free comparison class either (Malegiannaki et al., 2025). The relevant question is how factual content contributes to judgment incorporation and how the authority-source label contributes to authority deference within a given decision context.

This article introduces the Legal Evidence–Authority Dissociation (LEAD) framework for secondary analysis of legal judgment data. LEAD uses three consistent terms. The memory-entry gate concerns whether post-event information is recalled or misattributed to the trial. The judgment-incorporation gate concerns whether exposure to misinformation during deliberation (Study 1) or to different mitigating case facts (Studies 2 and 3) changes legal judgments. The authority-deference gate concerns whether a recommendation is weighted differently when it is attributed to a judge rather than an AI system. The gates are not assumed to be causally independent. They are separated because evidence for one gate does not establish another.

I evaluated LEAD using three public datasets with a combined retained sample of 1,922 participants. Study 1 reanalyzes jury-deliberation misinformation data and covers the memory-entry and judgment-incorporation gates (Cullen et al., 2024). Studies 2 and 3 reanalyze two sentencing experiments and cover the judgment-incorporation and authority-deference gates (Watamura et al., 2025). The reanalysis places outcomes on comparable within-study scales, while retaining raw units for interpretation. It then adds interval estimates, equivalence tests, ordinal models, and specification-curve checks. The framework provides a measurement and reporting structure for comparing existing outcomes. The design uses no synthetic observations and makes no claim about behavior in real courts.

## 2 Related work

### 2.1 Misinformation, source monitoring, and legal memory

Research on the misinformation effect has long shown that post-event information can alter what people later remember about an event. The effect is not limited to simple suggestibility. It involves source monitoring, accessibility, warnings, causal models, and the strength of the competing memory trace (Loftus, 2005; Jacoby, 1999; Ecker et al., 2010). Meta-analytic and experimental work indicates that warnings can reduce but rarely eliminate misinformation effects, especially when misinformation is accessible or plausibly integrated into the original event representation (Blank and Launay, 2014; Bulevich et al., 2022). These findings matter for legal judgment because jurors do not merely retrieve details. They must decide whether a remembered detail belongs to the trial evidence and whether it changes the legal conclusion.

Recent misinformation theory has moved away from treating false-memory reports as a single outcome. Continued-influence research separates belief persistence from source memory and highlights the role of explanatory coherence after correction (Lewandowsky et al., 2017; Ecker et al., 2022; Westbrook et al., 2023). Source-reliability work similarly shows that belief updating can be sensitive to who supplied information and how credible that source appears (Sanna and Lagnado, 2025). In the legal setting, this suggests a possible dissociation: a juror may remember or misattribute a detail without incorporating it into a verdict-relevant belief. The present reanalysis operationalizes that possibility by examining source-memory proportions, free-recall misinformation, and a composite legal-belief update in the same dataset.

The recent empirical literature reinforces why this operational separation is needed. Cross-cultural misinformation work indicates that post-event memory distortions are not confined to one legal culture or one participant population (Anakwah et al., 2024). Studies of fabricated lies and self-relevant false memory show that the perceived relation between a detail and the self or event history can change whether false information is later accepted as true (Rindal and Zaragoza, 2024; Wang et al., 2024). Yet these studies do not imply that every accepted false detail carries the same legal weight. A juror may misattribute a peripheral conversational detail to the trial record, whereas a different juror may reject the detail but still shift toward a prosecution-consistent story because the broader narrative has become more coherent.

Forensic and legal psychology also emphasizes that memory evidence is evaluated inside a social and procedural context. Jury instructions, deliberation, emotion, and group discussion can all alter how evidence is understood (Baguley et al., 2019; Salerno, 2021; Salerno and Diamond, 2010). Evidence can be biased by anchoring, early preferences, or coherence pressures before a final decision is made (Bystranowski et al., 2021; DeKay, 2015). These mechanisms make it risky to infer legal reliance from recall alone. A memory error is legally important when it enters the reasoning structure that supports guilt, credibility, liability, or punishment.

This point is central for intervention research. Judicial instructions, warnings, and expert explanations can be evaluated as memory safeguards, reasoning safeguards, or institutional legitimacy safeguards, but these are not the same endpoint. For example, a direction that reduces reliance on a rape myth may leave source memory unchanged, whereas a warning that improves source discrimination may leave the legal belief update unchanged. LEAD treats this as a design feature rather than a nuisance: interventions should be mapped to the gate they are intended to affect, and studies should avoid treating a single post-test judgment as evidence about all gates at once.

### 2.2 Evidence interpretation and coherence in legal proof

Legal proof is often discussed in normative terms, but empirical research shows that fact finders evaluate evidence through psychologically organized narratives. The story model proposes that jurors fit evidence into a causal structure, compare that structure with verdict categories, and select the best-fitting account (Pennington and Hastie, 1992). Later coherence-based accounts add that evidence evaluation and preference can mutually reinforce each other, producing systematic shifts before a final judgment is announced (Carlson and Russo, 2001; Simon and Read, 2023). In this view, the probative value of a fact is not only a property of the fact. It also depends on how the fact fits an evolving explanatory structure.

This matters because case content and an authority source can be confounded. A fact can change the legal story, whereas an authority-source label can be salient without adding probative information. Testimony is filtered through judgments about reliability, trust, and semantic fit (Harris et al., 2018; Gustafsson et al., 2024). Decision science also distinguishes literal information from gist-based interpretation and task-dependent weighting (Reyna and Brainerd, 2023; Summerfield and Parpart, 2022). LEAD applies this separation to legal psychology: an authority-source label is not equivalent to the facts it accompanies, and a remembered item is not equivalent to a legal update.

Other legal decisions show the same content-source tension under uncertainty. Child-custody decision makers report using cognitive strategies to make sense of uncertain and emotionally charged evidence, while plea-bargaining research shows that framing can alter legal choices even when the formal options are equivalent (Mendes and Ormerod, 2024; Reyna et al., 2025). These studies extend the content-source question to other legal settings and help locate the present contribution. Legal psychology needs constructs that travel across domains without collapsing distinct mechanisms into a broad bias label. LEAD offers one such construct by asking whether a finding belongs to memory entry, judgment incorporation, authority deference, or a combination of these processes.

### 2.3 AI advice, authority sources, and juror judgment

Algorithmic judgment research provides mixed expectations about judge-vs.-AI advice. Some studies find algorithm appreciation, especially when tasks appear objective or statistical (Logg et al., 2019). Others find algorithm aversion when tasks require empathy, moral sensitivity, or tolerance for error (Castelo et al., 2019; Rai and Diermeier, 2015). Trust in automated decision-making also depends on perceived legitimacy, fairness, and procedural acceptability (Araujo et al., 2020; Fine et al., 2025). Criminal sentencing combines these dimensions: it is legal, moral, emotionally salient, and institutionally constrained.

Watamura et al. (2025) directly tested whether jurors defer more to a human judge than to AI in criminal-trial vignettes. Their preregistered experiments crossed mitigating circumstances with conflicting recommendations from a judge and an AI system. The original article found that mitigation changed sentencing, whereas the authority-source main effects and interactions were non-significant. The present reanalysis compares the mitigation and authority-source coefficients in raw and standardized units. This comparison distinguishes magnitude from statistical significance.

Legal-AI research also cautions against interpreting an authority-source effect without the factual task. LegalBench and LawBench separate doctrinal, textual, and application-oriented performance, and step-by-step legal reasoning remains difficult for large language models (Guha et al., 2023; Fei et al., 2024; Mishra et al., 2025). A juror-facing vignette asks a different question: whether attributing the prison recommendation to a judge rather than AI changes the participant's judgment. Public-perception research suggests that legitimacy and trust may shape this response (Fine et al., 2025; Iannuzzi et al., 2025). LEAD therefore embeds the authority-source manipulation within the case-fact manipulation.

### 2.4 Transparent inference for small or null effects

Because LEAD depends on dissociation, it requires methods that do not turn every non-significant test into evidence for absence. Equivalence testing asks whether an effect falls within a declared negligible range (Lakens et al., 2018). Statistical guidance also stresses that null-hypothesis tests and estimation answer different questions (Pawel et al., 2024; Kiers and Tendeiro, 2025; Schubert et al., 2025). The present analysis therefore reports confidence intervals and two-one-sided equivalence tests for the authority-source effect.

Model choice also matters. Sentencing ratings are ordinal, although metric models are often used for multi-point scales (Liddell and Kruschke, 2018). This article therefore compares robust ordinary least squares with ordinal logit models. A specification curve also shows how the authority-source estimate changes across defensible choices (Steegen et al., 2016; Simonsohn et al., 2020). Together, these analyses evaluate whether the conclusion depends on one model or one equivalence boundary.

This inferential stance is especially important for secondary analysis. Guidance on statistical reporting separates estimation, null-hypothesis testing, and practical conclusions (Kiers and Tendeiro, 2025; Schubert et al., 2025). Absence claims require intervals and design sensitivity, not only a large *p* value (Pawel et al., 2024). A non-significant authority-source coefficient is therefore reported with its interval, equivalence test, and boundary sensitivity.

## 3 Materials and methods

### 3.1 Data sources and inclusion

All analyses used public, de-identified secondary data. No new participants were recruited, and no synthetic observations were added. The jury-deliberation data came from the public OSF repository for Cullen et al. (2024) (https://osf.io/wqgsm/). The source article reports a retained Study 1 sample of 275 participants after exclusions. The present project used the authors' main SPSS dataset, source-memory dataset, and analysis scripts for Study 1. These are individual mock-juror responses, not independent decisions made by a completed jury: each participant made the pre- and post-deliberation judgments, and the two computer-controlled jurors supplied the scripted deliberation. We therefore use “participant” or “mock juror” for these observations rather than treating them as group jury decisions. The source article also links a Study 2 repository, but that node was not anonymously accessible during this project; therefore the source article's Study 2 was not represented as an analyzed dataset.

The judge-vs.-AI data came from the public OSF repository for Watamura et al. (2025) (https://osf.io/n6tm2/). The present analyses used the two Excel workbooks corresponding to the source article's Experiment 1 and Experiment 2; throughout this manuscript, these are called Study 2 and Study 3, respectively. Following the source study's attention-check exclusions, the retained samples were 787 and 860 participants. Full pre-exclusion samples were retained only for sensitivity analyses. Table 1 summarizes the analytic datasets.

**Table 1:** Datasets and primary analytic roles.

Dataset	Legal setting	Gates measured in LEAD	Retained sample
Jury misinformation Study 1	Alleged sexual assault trial and simulated jury deliberation	Memory-entry and judgment-incorporation gates	275
Judge-vs.-AI Study 2 (source Experiment 1)	Murder sentencing with intimate-partner violence mitigation	Judgment-incorporation and authority-deference gates	787
Judge-vs.-AI Study 3 (source Experiment 2)	Murder sentencing with caregiver-burden mitigation	Judgment-incorporation and authority-deference gates	860

Raw files were stored separately from processed tables. SHA-256 checksums were generated for every raw file, and the cleaning script wrote processed CSV files without modifying the raw data. Analyses were run in Python using pandas, statsmodels, numpy, scipy, matplotlib, and seaborn. The source data links are reported in the main text and in the Data Availability Statement.

The datasets were selected for substantive fit and reproducibility. Together, they cover three outcomes without merging them into one model. Study 1 measures memory entry and judgment incorporation. Studies 2 and 3 measure judgment incorporation through sentencing and authority deference through the judge-vs.-AI manipulation. Judgment incorporation is therefore the overlapping gate across the two data streams. LEAD compares the gate-specific estimates; it does not assume that each dataset represents a unique gate.

### 3.2 The LEAD framework

LEAD defines three gates using observed outcomes. The memory-entry gate, *G*_1_, asks whether post-event information is treated as part of the trial record:


$$\begin{array}{l} G_{1}=P\left(\text{source confusion}|\text{post}\text{-event information}\right). \end{array}$$ (1)

Equation 1 was estimated with the proportion of misinformation items misattributed to the trial and with counts of accepted misinformation in free recall. In the jury misinformation data, this was estimated with the proportion of misinformation items misattributed to the trial and with counts of accepted misinformation in free recall.

The judgment-incorporation gate, *G*_2_, asks whether a legal judgment differs with the content presented to participants. Its general definition is a contrast between evidence-content conditions:


$$\begin{array}{l} G_{2}\left(e,e_{0}|X\right)=𝔼\left(J^{*}|e,X\right)-𝔼\left(J^{*}|e_{0},X\right). \end{array}$$ (2)

Here J^*^ is the study-specific judgment outcome, *e* and *e*_0_ are the compared content conditions, and *X* denotes covariates when included. In Study 1, J^*^ is the directional legal-belief update (LBU), calculated from pre-to-post changes in guilt rating and complainant credibility:


$$\begin{array}{l} LBU_{i}=\frac{z\left(\Delta \text{guil}{\text{t}}_{i}\right)+z\left(\Delta \text{credibilit}{\text{y}}_{i}\right)}{2}. \end{array}$$ (3)

Equation 3 defines the participant-level LBU outcome. Positive values indicate movement toward a more prosecution-consistent judgment. Each change is the post-deliberation rating minus its pre-deliberation rating. Here *z*(Δ) denotes division by that change score's within-study sample standard deviation, preserving zero as no change. The two scaled changes are averaged to produce one LBU outcome per participant.

For Study 1, Equation 2 compares mean LBU in the pro-prosecution or contradictory misinformation condition (*e*) with mean LBU in the pro-defense condition (*e*_0_). Thus, the outcome variable is LBU and the explanatory variable is misinformation condition, entered as two indicators with pro-defense as the reference. The primary LBU model includes no additional covariates (*X* is empty); baseline ratings enter through the change scores. Separate component models regress post-deliberation guilt or credibility on its corresponding baseline rating and misinformation condition.

For Studies 2 and 3, Equation 2 uses J^*^ = *Y*, the single post-vignette sentencing response, with mitigating circumstances present (*e*) vs. absent (*e*_0_). Equation 5 estimates this contrast using mitigation, authority-source assignment, their interaction, age, and sex. At a fixed centered authority assignment *A*, the adjusted mitigation contrast is β_*E*_+β_*EA*_*A*. Averaging equally over the two authority assignments (*A* = −0.5 and *A* = 0.5) yields β_*E*_, the reported G2 estimate. These studies therefore operationalize G2 through a between-condition sentencing contrast; LBU is specific to Study 1.

The authority-deference gate, *G*_3_, asks whether reliance changes when the prison recommendation is attributed to a judge rather than an AI system:


$$\begin{array}{lll} G_{3} & = & E\left(Y|\text{judge recommends prison}\right) \end{array}$$

$$\begin{array}{l} -\;E\left(Y|\text{AI recommends prison}\right). \end{array}$$ (4)

Equation 4 defines the authority-source contrast. Here *Y* was the 1–8 sentencing response, where higher values indicate a more prison-oriented judgment. LEAD does not assume that *G*_1_, *G*_2_, and *G*_3_ are causally independent. It requires each gate to be measured before a claim is made about it.

### 3.3 Study 1 variables and models

Study 1 used a one-factor, three-condition design (Cullen et al., 2024). Participants read a 1,534-word transcript of an alleged alcohol-involved acquaintance sexual assault. Before deliberation, they gave a guilty or not-guilty verdict, rated guilt from 1 to 7, and rated the complainant's honesty, believability, credibility, and accuracy. They then entered a text-only simulated deliberation with two computer-controlled jurors. The analyses below use the participant's individual judgments; the scripted jurors were part of the misinformation-exposure manipulation and were not treated as additional independent observations.

The deliberation supplied four false details in one of three forms. In the consistent pro-prosecution condition, both simulated jurors supplied the same prosecution-favoring detail. For example, they said that the complainant and defendant had known each other for two weeks, although the transcript stated 4 weeks. In the consistent pro-defense condition, both supplied the same defense-favoring alternative, such as six weeks. In the contradictory condition, one simulated juror supplied the two-week detail and the other supplied the 6-week detail. The same structure was used for relationship status, drinks consumed, and the complainant's reaction to touching.

After deliberation, participants repeated the verdict, guilt, and credibility measures. They then completed a three-minute free-recall task and a 24-item source-memory test. The source test required each detail to be classified as appearing in the trial only, deliberation only, both, or neither. The primary memory-entry outcome was the participant-level proportion of misinformation items incorrectly attributed to the trial. This bounded proportion was modeled with fractional logit and robust standard errors. Free-recall misinformation counts were modeled with Poisson regression. Pro-defense misinformation was the reference condition.

The judgment-incorporation outcome in the primary model was LBU, the mean of the two scaled pre-to-post changes in guilt and complainant credibility. Positive values indicated movement toward the prosecution. The model regressed LBU on two misinformation-condition indicators (pro-prosecution and contradictory), with pro-defense as the reference. Each condition coefficient estimates the corresponding mean-LBU contrast in Equation 2; HC3 robust standard errors were used. Component checks regressed post-deliberation guilt or credibility on the corresponding baseline rating and the same condition indicators. The association between memory intrusion and judgment incorporation was estimated by regressing LBU on free-recall misinformation count and condition. Bootstrap intervals based on 4,000 draws were used for selected contrasts and suspicious-participant sensitivity checks.

### 3.4 Studies 2 and 3 variables and models

Studies 2 and 3 were separate 2-by-2 online experiments conducted in the context of Japan's lay-judge system (Watamura et al., 2025). In the remainder of the manuscript, “Study 2” means the source article's Experiment 1 and “Study 3” means its Experiment 2. Participants were asked to decide between prison and a suspended sentence. They were told that an experienced judge and an AI trained on case law had received the same defendant background, criminal record, and mitigating information. The experimental materials presented two conflicting recommendations attributed to the judge and AI.

Study 2 (source Experiment 1) varied whether a woman killed her husband after sustained domestic violence or because she was tired of living with his chronic illness. Study 3 (source Experiment 2) varied whether a man killed his terminally ill mother after she wanted to die or because he resented pressure to leave the house. Each study crossed two mitigation conditions with two authority assignments, yielding four experimental cells. In one authority assignment, the judge recommended prison and the AI recommended suspension; in the other, the AI recommended prison and the judge recommended suspension. Both assignments occurred within each mitigation condition. The primary analyses included retained participants from all four cells in each study. Participants then reported their own sentence on an eight-point scale, from 1 (suspended sentence) to 8 (prison sentence), and completed manipulation and attention checks.

The first factor therefore changed legally relevant case content: mitigating circumstances present vs. absent. The second changed the authority-source assignment: judge recommends prison vs. AI recommends prison. The primary model for each study was


$$\begin{array}{l} Y_{i}=\alpha +\beta_{E}E_{i}+\beta_{A}A_{i}+\beta_{EA}E_{i}A_{i}+\gamma_{1}\text{Ag}{\text{e}}_{i}+\gamma_{2}\text{Se}{\text{x}}_{i}+\epsilon_{i}, \end{array}$$ (5)

where *Y*_*i*_ is the participant's 1–8 sentencing response. Mitigation is coded *E*_*i*_ = 0.5 when present and *E*_*i*_ = −0.5 when absent. Authority assignment is coded *A*_*i*_ = 0.5 when the judge recommends prison and *A*_*i*_ = −0.5 when the AI recommends prison. The product *E*_*i*_*A*_*i*_ represents their interaction; age is measured in years and sex is coded 1 for female and 0 otherwise. The coefficient β_*E*_ estimates judgment incorporation as defined in Equation 2, averaged equally across the two authority assignments. The coefficient β_*A*_ estimates authority deference, averaged equally across the two mitigation conditions. HC3 robust standard errors were used for the primary ordinary least squares models.

Because the sentencing outcome is ordinal, ordered logit models were used as a model-family sensitivity analysis: they test whether the direction and inferential status of the authority-source result depend on treating the 1–8 response as metric. Full pre-exclusion samples were analyzed as an inclusion sensitivity analysis: they show whether the result changes materially when the source article's attention-check exclusions are not applied. The specification curve is a decision-sensitivity analysis: it shows how the standardized authority-source estimate changes across 16 prespecified combinations of study, sample, covariate adjustment, and interaction choice. These analyses do not estimate statistical power or the effect a sample could detect. A random-effects synthesis combined the two authority-source coefficients while allowing between-study heterogeneity. This synthesis was descriptive because only two studies were available.

The mitigation and authority-source coefficients were standardized within each study after the primary models were estimated. This placed both manipulations in standard-deviation units for direct comparison. It did not combine participants, outcomes, or studies. Raw coefficients on the 1–8 sentencing scale were retained for interpretation. Every central result is therefore reported in raw points, standardized units, confidence intervals, and *p* values.

### 3.5 Equivalence and specification-curve analyses

The authority-source effect was evaluated with two-one-sided tests (TOST). The main smallest effect size of interest was set to |*d*| = 0.20, a conventional small-effect boundary. Equivalence was concluded only when the 90% confidence interval fell completely inside [−0.20, 0.20]. TOST analyses were repeated for |*d*| = 0.10, 0.15, 0.25, and 0.30 to show boundary sensitivity. Thus, TOST asks whether the source effect is within a declared small-effect range; it is not the same as a non-significance test.

Specification-curve analyses varied study, sample definition, covariate adjustment, and inclusion of the mitigation-by-authority-source interaction. Each specification estimated the standardized authority-source coefficient. The goal was to show the range under defensible analytic choices, not to search for significance. Taken together, the six robustness/inference components used here serve distinct roles: robust OLS protects the primary standard errors against heteroskedasticity; ordered logit checks the ordinal outcome scale; full-sample analysis checks exclusion sensitivity; the specification curve checks analytic-choice sensitivity; random-effects pooling summarizes cross-study direction descriptively; and TOST tests compatibility with the prespecified small-effect bounds.

## 4 Results

### 4.1 The LEAD framework organized three separable gates

Figure 1 shows the analytic pipeline used for the reanalysis. The two input streams are first checked, coded, and standardized within study. Stream 1 (the jury-misinformation dataset) supplies memory-entry and judgment-incorporation outcomes; Stream 2 (Study 2, source Experiment 1, and Study 3, source Experiment 2) supplies judgment-incorporation and authority-deference outcomes. The arrows in the original pipeline indicate processing and measurement coverage, not causal effects between gates. Panel C conceptually compares estimates from the study-specific models: the memory-entry estimates are obtained from Study 1, whereas β_*E*_ and β_*A*_ are obtained from the sentencing model in Studies 2 and 3. The effect-decomposition, robustness, and bounded-output modules therefore receive the estimates produced by the relevant gates, while the real-court boundary remains a scope condition for interpretation.

**Figure 1:**
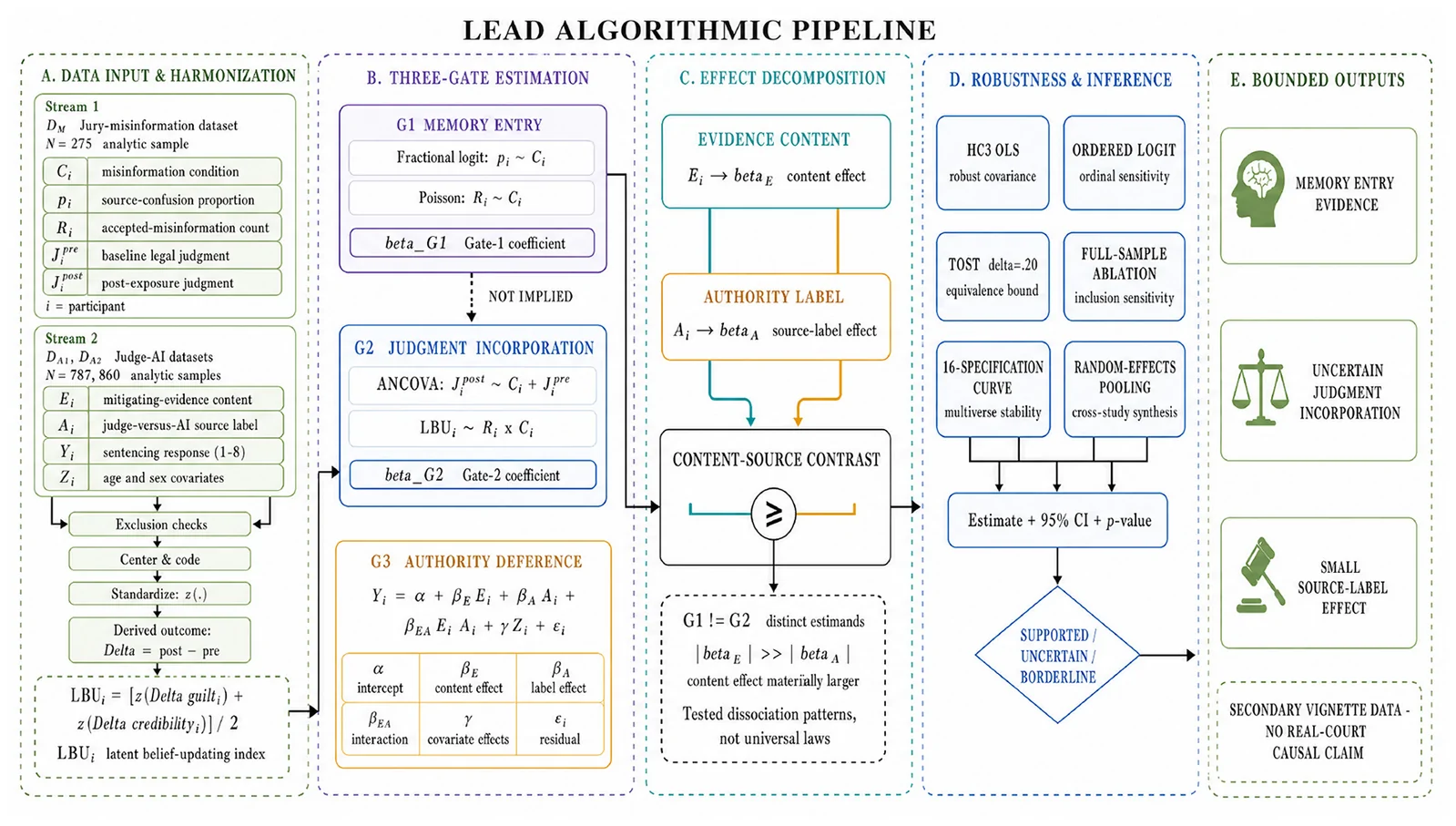
Legal Evidence–Authority Dissociation (LEAD) analytic pipeline (original submission figure retained unchanged). Stream 1 is the jury-misinformation dataset and Stream 2 contains Study 2 (the source article's Experiment 1) and Study 3 (its Experiment 2). After exclusion checks, coding, standardization, and construction of the directional legal-belief update, the pipeline estimates memory entry (G1), judgment incorporation (G2), and authority deference (G3). Stream 1 contributes G1 and the pre-to-post version of G2; Stream 2 contributes the case-content version of G2 (β_*E*_) and G3 (β_*A*_). The pre-to-post G2 notation in Panel B illustrates Study 1; Equation 2 defines the general condition contrast, implemented for Stream 2 through Equation 5. Panel C conceptually compares these separately estimated gate-specific effects. The arrows show processing and measurement coverage rather than causal flow or predictive claims. Robustness and inference modules report confidence intervals, *p* values, equivalence, and sensitivity; the final panel states bounded conclusions derived from the examined studies.

The five panels have distinct jobs. Panel A lists the observed inputs and derived variables: source confusion, free-recall misinformation, pre- and post-deliberation judgments, mitigation content, authority-source assignment, and sentencing response. Panel B shows the three gate estimands. Its G1 and pre-to-post G2 quantities apply to Study 1. The displayed G2 notation illustrates the pre-to-post setting; Equation 2 specifies the general condition contrast and its study-specific implementation. For Studies 2 and 3, G2 is β_*E*_ in Equation 5, andG3 is β_*A*_. Panel C compares the separately estimated Study 1 memory-entry effects and Studies 2–3 evidence-content and authority-source effects. Panel D shows the six complementary inference checks described below. Panel E summarizes the bounded conclusions supported by the relevant gate estimates and inference checks.

### 4.2 Misinformation entered source memory more clearly than legal belief

The jury misinformation data showed evidence for memory-entry effects. The mean proportion of misinformation items misattributed to the trial was 0.13 in the pro-defense condition, 0.16 in the contradictory condition, and 0.27 in the pro-prosecution condition. In the fractional-logit model, the pro-prosecution condition increased source-confusion log odds relative to the pro-defense condition, *b* = 0.961, 95% CI [0.462, 1.460], *p* < 0.001. The contradictory condition was not clearly different from the pro-defense condition, *b* = 0.242, 95% CI [-0.249, 0.732], *p* = 0.334. Thus, the clearest memory-entry contrast was pro-prosecution vs. pro-defense misinformation.

Free recall showed a different pattern. The mean number of accepted misinformation items was 0.29 in the pro-defense condition, 0.14 in the contradictory condition, and 0.45 in the pro-prosecution condition. The contradictory condition produced fewer accepted items than the pro-defense condition, *b* = −0.748, 95% CI [-1.468, -0.028], *p* = 0.042. The pro-prosecution vs. pro-defense contrast was not statistically significant, *b* = 0.422, 95% CI [-0.179, 1.023], *p* =.169. Source attribution and free recall therefore did not show identical condition contrasts.

The judgment-incorporation contrasts were not statistically significant. The pro-prosecution vs. pro-defense contrast was 0.028 SD-composite units, 95% CI [-0.236, 0.292], *p* = 0.834. The contradictory vs. pro-defense contrast was -0.021, 95% CI [-0.280, 0.238], *p* = 0.874. The separate guilt and credibility models were also non-significant. In Figure 2, Panel A shows the higher source-misattribution distribution for pro-prosecution misinformation. Panel B shows substantial overlap in legal-belief updating. Panel C shows the weak within-condition associations between free-recall misinformation and legal-belief updating, and Panel D shows overlap in the joint guilt and credibility changes.

**Figure 2:**
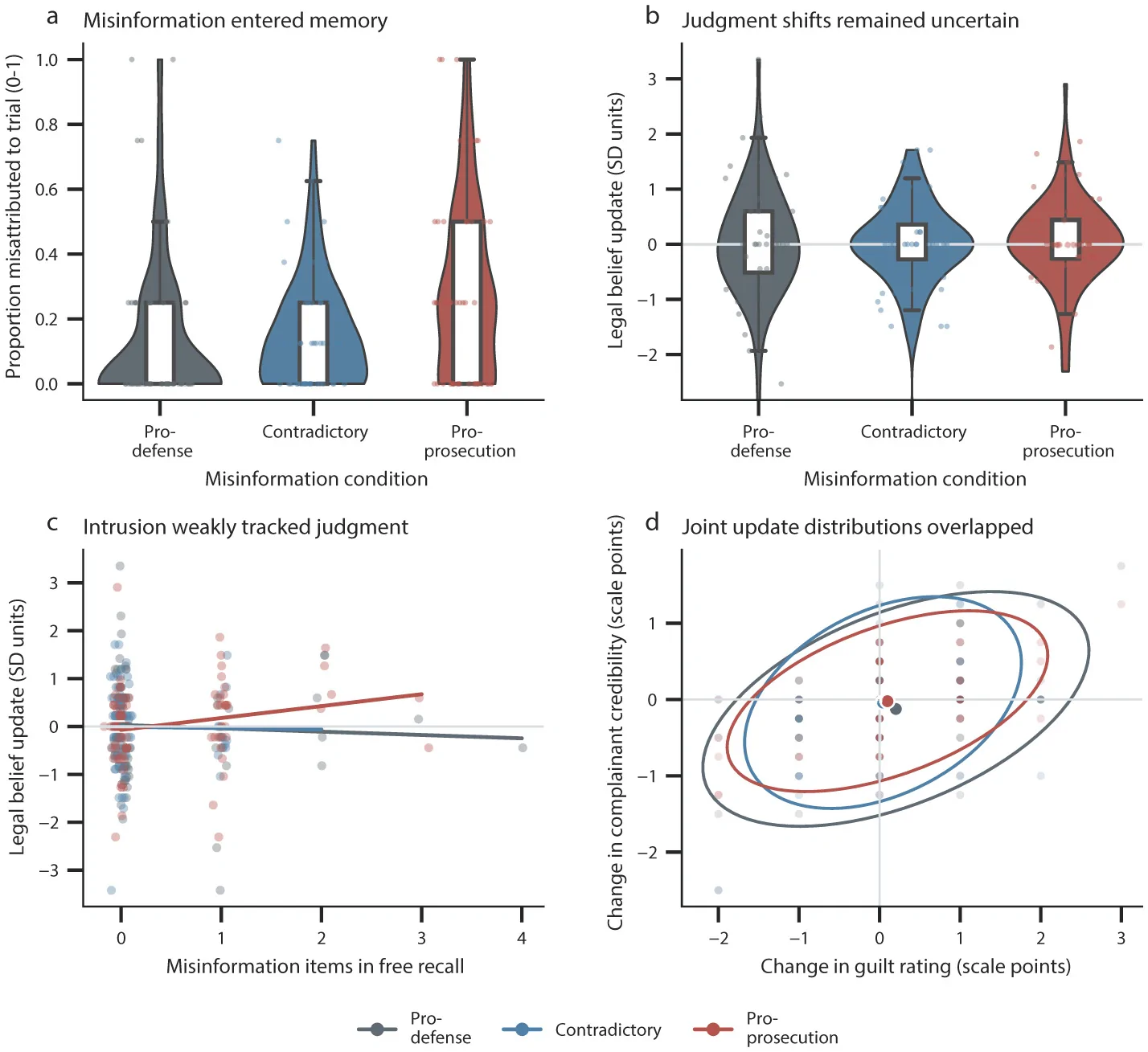
Memory-entry and judgment-incorporation outcomes in Study 1. **(A)** Participant-level proportions of misinformation items misattributed to the trial. **(B)** Directional legal-belief update distributions; zero indicates no pre-to-post change. In A and B, violins show distributions, boxes show medians and interquartile ranges, and points are sampled observations displayed to reduce overplotting. **(C)** Accepted misinformation in free recall vs. legal-belief updating. Points are participants with horizontal jitter, and colored lines are unadjusted within-condition least-squares fits. **(D)** Participant-level changes in guilt and complainant credibility. Small points are participants, large points are condition means, and ellipses are 95% covariance contours.

The association between free-recall misinformation and LBU was also weak. The adjusted coefficient for accepted misinformation items was 0.070 SD-composite units per item, 95% CI [-0.098, 0.238], *p* = 0.411. This does not demonstrate that memory intrusion is irrelevant to legal belief. It indicates that, in this dataset and model, memory intrusion was not a stable predictor of the composite legal update. The appropriate conclusion is therefore a bounded dissociation: misinformation more clearly crossed the memory-entry gate than the judgment-incorporation gate.

### 4.3 Case facts outweighed the authority-source manipulation

The sentencing experiments showed large mitigation effects. In Study 2 (source Experiment 1), mitigating facts reduced sentencing by 1.763 points on the 1–8 scale, 95% CI [-2.079, -1.446], *p* < 0.001. In Study 3 (source Experiment 2), the reduction was 2.367 points, 95% CI [-2.642, -2.092], *p* < 0.001. These estimates corresponded to standardized effects of approximately *d* = −0.75 and *d* = −1.01. Robust ordinary least squares and ordinal logit models gave the same direction.

The authority-source coefficients were much closer to zero and were not statistically significant. In Study 2 (source Experiment 1), the adjusted coefficient was 0.036 sentencing points, 95% CI [−0.278, 0.351], *p* = 0.822, corresponding to *d* = 0.015. In Study 3 (source Experiment 2), it was -0.238 points, 95% CI [-0.512, 0.037], *p* = 0.090, corresponding to *d* = −0.102. The mitigation-by-authority-source interactions were also non-significant in Study 2, *b* = 0.214, 95% CI [-0.414, 0.842], *p* = 0.504, and Study 3, *b* = 0.111, 95% CI [-0.440, 0.661], *p* =.694. Panels A and B of Figure 3 show the four experimental conditions. Panel C places the mitigation and authority-source estimates on the same standardized scale, and Panel D shows their confidence intervals relative to the equivalence region.

**Figure 3:**
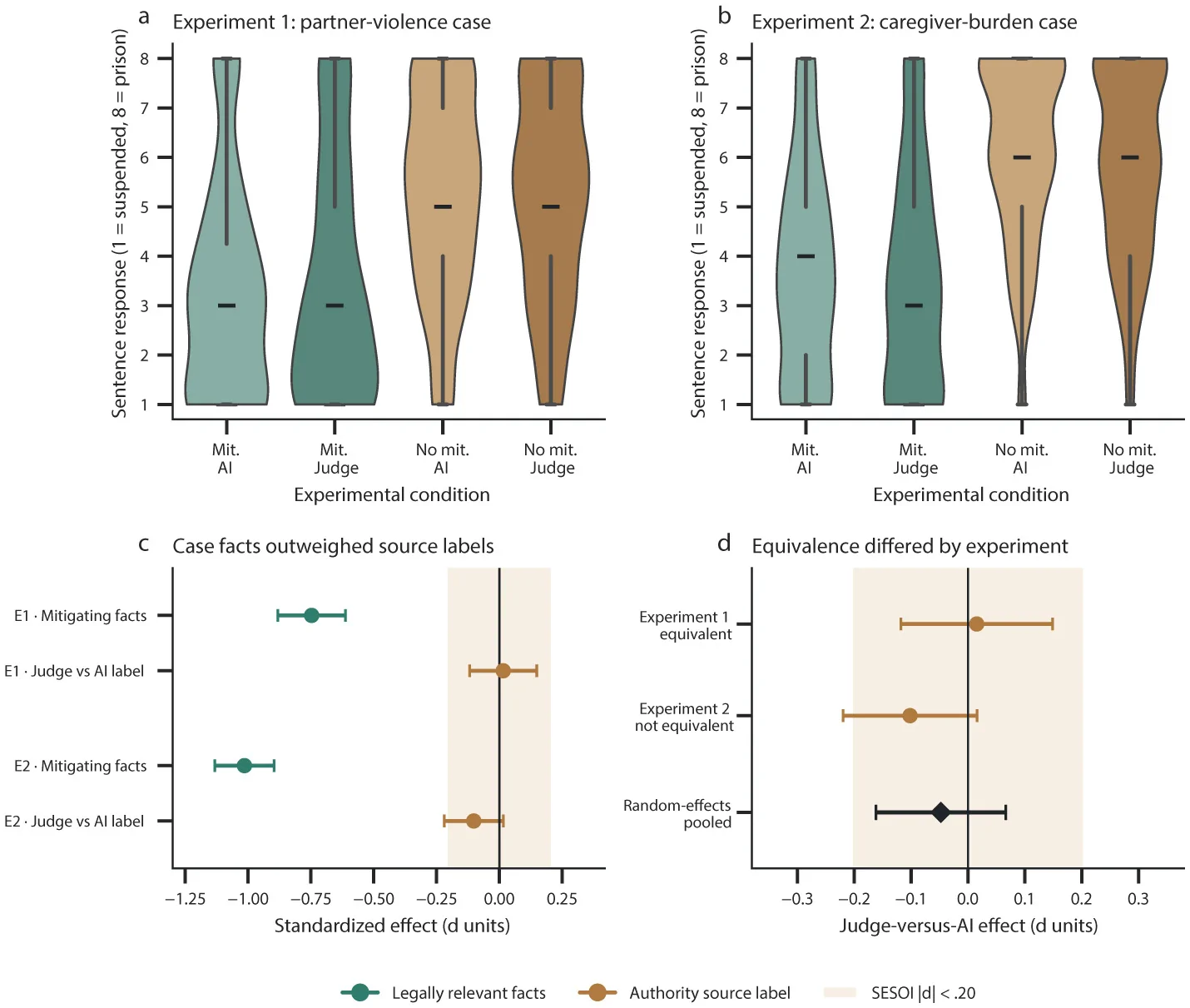
Judgment-incorporation and authority-deference outcomes in Studies 2 and 3 (the source article's Experiments 1 and 2). The original figure labels retain the source article's Experiment 1/2 notation. **(A, B)** Sentencing distributions for the four combinations of mitigating circumstances and the authority recommending prison in Studies 2 and 3. Violins show participant distributions and boxes show medians and interquartile ranges. **(C)** Standardized mitigation and authority-source coefficients with 95% confidence intervals. **(D)** Authority-source coefficients with 95% confidence intervals; the shaded band marks the prespecified |*d*| < 0.20 equivalence region. Equivalence tests use 90% confidence intervals and are reported in the text.

The random-effects synthesis of the two authority-source estimates produced a pooled coefficient of -0.112 sentencing points, 95% CI [-0.380, 0.156], *p* = 0.412, with τ^2^ = 0.0148. Because only two experiments were available, this synthesis is descriptive. It should not be treated as a stable population estimate of judge-vs.-AI deference.

### 4.4 Equivalence evidence was asymmetric across studies 2 and 3

TOST analyses clarified what the non-significant authority-source tests could support. In Study 2 (source Experiment 1), the standardized estimate was *d* = 0.015, and its 90% confidence interval fell within the declared |*d*| = 0.20 equivalence bounds. The TOST *p* value was 0.003, supporting equivalence at that boundary. In Study 3 (source Experiment 2), the estimate was *d* = −0.102, but its 90% interval did not fall completely within the same bounds; the TOST *p* value was 0.051. The second result is therefore inconclusive at |*d*| = 0.20, not evidence of equivalence.

The declared smallest effect size of interest therefore mattered. Study 2 met equivalence criteria at |*d*| = 0.15 and larger, but not at |*d*| = 0.10. Study 3 met equivalence criteria at |*d*| = 0.25 and 0.30, but not at |*d*| = 0.20 or smaller. The estimates were small relative to the mitigation effects, but only Study 2 established equivalence at the prespecified boundary.

### 4.5 Specification and model-family checks supported the main pattern

The specification curve covered 16 defensible authority-source models. In Figure 4A, each point is one standardized coefficient with its 95% confidence interval. The matrix below the points identifies the study, retained or full sample, covariate set, and interaction choice. All point estimates fell within or close to the |*d*| = 0.20 region. Study 3 retained-sample estimates were more negative than those from Study 2, but none approached the standardized mitigation effects.

**Figure 4:**
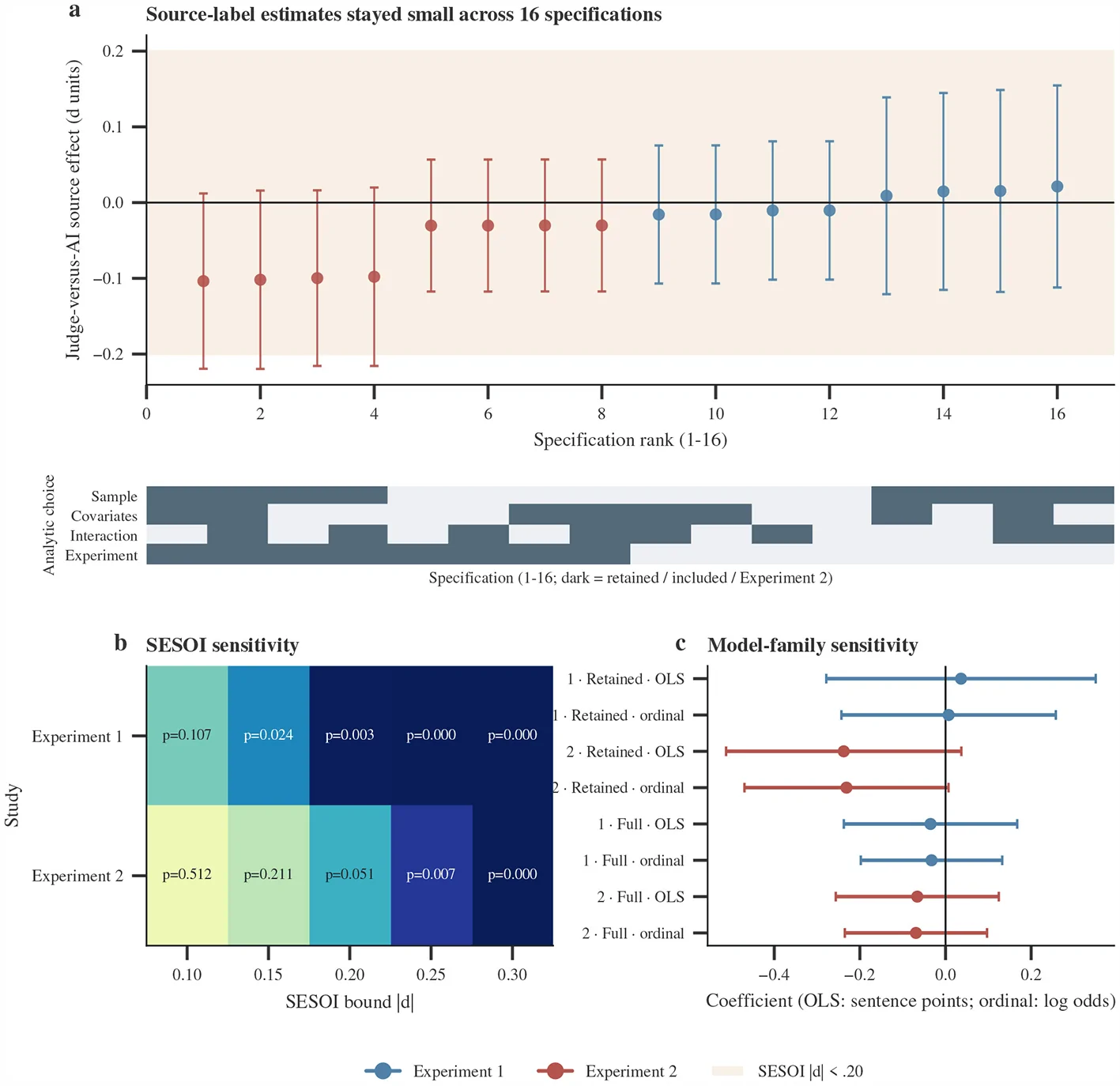
Robustness and sensitivity analyses for authority-source effects in Studies 2 and 3. **(A)** Sixteen standardized coefficients with 95% confidence intervals across study, sample, covariate, and interaction choices. The matrix identifies each analytic choice; the shaded band marks |*d*| < 0.20. **(B)** TOST *p* values across smallest-effect-size-of-interest bounds. Darker cells indicate smaller *p* values, and equivalence requires *p* < 0.05. **(C)** Authority-source coefficients and 95% confidence intervals from ordinary least squares and ordinal logit models in retained and full samples. Coefficients remain on their respective model scales and are compared by direction and inferential conclusion, not numerical magnitude.

The ordinal models produced the same directions as ordinary least squares. The ordered-logit authority-source coefficient was 0.007 in Study 2, 95% CI [-0.243, 0.258], *p* = 0.955, and -0.231 in Study 3, 95% CI [-0.470, 0.007], *p* = 0.057. Full-sample analyses were also non-significant. Figure 4B reports TOST *p* values across five equivalence bounds: darker cells indicate stronger evidence for equivalence. Panel C compares retained and full samples under metric and ordinal models. These checks preserve uncertainty in Study 3 rather than converting a non-significant test into evidence of absence. Figure 4C compares retained and full samples under metric and ordinal models.

## 5 Discussion

This multistudy reanalysis supports the central LEAD claim: memory entry, judgment incorporation, and authority deference should be measured separately in legal psychology. In Study 1, pro-prosecution misinformation was more likely than pro-defense misinformation to be misattributed to the trial record, but the corresponding legal-belief contrasts were non-significant. In Studies 2 and 3, mitigating facts produced large sentencing shifts, whereas the authority-source coefficients were non-significant and close to zero. Study 2 established equivalence at |*d*| = 0.20, but Study 3 did not. The evidence therefore supports a bounded dissociation, not causal independence among the gates.

The reanalysis extends the source articles by changing the comparison and the inferential question, not by replacing their conclusions. Table 2 separates the original findings from the added LEAD analysis. The jury study originally compared three misinformation conditions on memory and participant-level legal judgments. LEAD retains those outcomes but places the pre-to-post guilt and credibility changes on a common directional scale and directly compares the memory-entry and judgment-incorporation patterns. The sentencing article originally tested main and interaction effects in two experiments. LEAD retains that design, compares mitigation and authority-source coefficients in raw and standardized units, and asks whether the authority-source effects are small enough to meet declared equivalence bounds. LEAD is therefore a measurement and reporting framework. The present analyses do not test whether LEAD predicts individual judgments better than a competing model or a single composite score.

**Table 2:** Original study conclusions and the additional contribution of the LEAD reanalysis.

Source dataset	Original conclusion	Added LEAD contribution
Jury misinformation Study 1	Misinformation condition affected memory measures, especially for consistent pro-prosecution misinformation, but did not significantly affect post-deliberation legal decisions.	Separates memory entry from judgment incorporation, builds a directional pre-to-post legal-belief composite, and estimates whether recalled misinformation covaries with legal-belief change.
Judge-vs-AI Study 2 (source Experiment 1)	Mitigating circumstances affected sentencing; judge-vs.-AI recommendation pattern and its interaction with mitigation were non-significant.	Reports mitigation and authority-source coefficients in raw and standardized units, adds confidence intervals, equivalence tests, ordinal models, and specification checks.
Judge-vs-AI Study 3 (source Experiment 2)	The mitigation result replicated; authority-source and interaction tests remained non-significant.	Shows that the authority-source estimate is small relative to mitigation but does not meet the prespecified |*d*| = 0.20 equivalence criterion, preserving the remaining uncertainty.

The first implication concerns misinformation safeguards. It would be tempting to treat source confusion as sufficient evidence that jurors relied on misinformation. The present analysis argues against that shortcut. Source misattribution is legally serious, but its inferential role depends on whether the information changes the judgment structure. This distinction also clarifies why warnings, instructions, and source-monitoring interventions should be evaluated on multiple endpoints rather than on memory accuracy alone (Blank and Launay, 2014; Ecker et al., 2022; Baguley et al., 2019). A safeguard that improves source monitoring may still leave legal interpretation unchanged, and a safeguard that reduces belief updating may not eliminate memory contamination.

The second implication concerns AI advice in legal settings. Studies 2 and 3 did not show statistically significant authority-source effects. They also do not prove that jurors treat judges and AI systems identically in other contexts. The experimental “AI” was a label attached to a conflicting recommendation, not a deployed model that processed a transcript. Participants were told only that both authorities received the same background, criminal-record, and mitigating information. The result therefore concerns responses to an authority-source assignment under fixed case information. Transparency, legitimacy, empathy, and procedural justice require separate manipulations (Araujo et al., 2020; Fine et al., 2025; Watamura et al., 2023).

This finding is not an endorsement of AI advice in courts. Court-facing tools range from research and administration to prediction and decision support, with different inputs and risks (European Commission for the Efficiency of Justice, 2018). Criminal-risk systems can also reproduce limitations in their training and input variables (Dressel and Farid, 2018). The present analysis makes a narrower point: mitigating facts shifted sentencing much more than the authority-source assignment in these vignettes. Claims about deployment require direct tests of transparency, explanation quality, error history, data inputs, and procedural safeguards.

The third implication is methodological. Non-significance and smallness are different conclusions. Both authority-source coefficients were non-significant, but only Study 2 met the prespecified equivalence criterion. Study 3 remained inconclusive at that boundary. Specification-curve and ordinal-model checks showed that this distinction persisted across reasonable choices (Steegen et al., 2016; Simonsohn et al., 2020; Liddell and Kruschke, 2018). Reporting estimates, intervals, conventional tests, and equivalence tests prevents a null-hypothesis result from carrying more meaning than it supports.

Several limitations constrain the conclusions. First, all analyses are secondary, and the source studies were not designed to test LEAD prospectively. The analyses cannot identify causal pathways among memory, evidence interpretation, and authority deference. Second, the scenarios were online vignette or mock-jury tasks without real legal consequences. Third, the inaccessible second misinformation dataset prevented replication of the memory-judgment comparison. Fourth, Studies 2 and 3 were conducted in Japan's lay-judge context. Authority-source effects may differ where judges, jurors, or automated tools have different roles. Fifth, the AI manipulation supplied a label and recommendation but no explanation, uncertainty estimate, interface, or observable model process. The findings do not generalize to systems that analyze transcripts, filings, or structured risk data.

Future work should test LEAD prospectively. A stronger design would independently manipulate evidentiary content, source reliability, and authority source while measuring source memory, narrative coherence, confidence, and legal judgment over time. This design could distinguish memory-mediated from interpretation-mediated effects and test whether deliberation changes authority deference. Legal AI studies could compare judges, AI systems, expert witnesses, and peer jurors under identical factual content. Case content and authority source should be separated by design.

A prospective LEAD study would also clarify where legal expertise enters the chain. Legal professionals may recognize some memory hazards but still use heuristics under uncertainty. Lay jurors may respond differently when AI advice includes an explanation rather than a bare authority-source label (Houben et al., 2024; Malegiannaki et al., 2025). A mock trial could combine misinformation with an authority-source manipulation and preregistered equivalence bounds. It could then test whether authority matters more when the factual record is ambiguous.

## 6 Conclusion

The present reanalysis shows that source memory, legal-belief updating, and authority-source effects had different empirical profiles in three public datasets. Compared with the original study-level analyses, LEAD makes two cross-gate comparisons explicit: in Study 1, memory-entry evidence was clearer than judgment-incorporation evidence, and in Studies 2 and 3, case-content effects were much larger than authority-source effects. LEAD does not claim to predict individual judgments better than an untested alternative. Its immediate value is methodological: researchers should identify which gate an intervention measures and should not infer memory entry, judgment incorporation, or authority deference from one another without direct evidence.

## Data Availability

The public datasets analyzed in this study are available at OSF: jury-deliberation misinformation Study 1, https://osf.io/wqgsm/; judge-versus-AI Studies 2–3 (source Experiments 1–2), https://osf.io/n6tm2/. Processed tables, analysis outputs, figure scripts, and checksum records are included with the submitted project materials.
